# Molecular Characterization of* Salmonella* from Human and Animal Origins in Uganda

**DOI:** 10.1155/2017/4604789

**Published:** 2017-05-28

**Authors:** Atek Atwiine Kagirita, Andrew Baguma, Tonny Jimmy Owalla, Joel Bazira, Samuel Majalija

**Affiliations:** ^1^Department of Disease Surveillance and Outbreak, Uganda National Health Laboratories Services, Ministry of Health, P.O. Box 7272, Kampala, Uganda; ^2^Department of Microbiology, Faculty of Medicine, Mbarara University of Science and Technology, P.O. Box 1410, Mbarara, Uganda; ^3^Division of Infectious Diseases, Med Biotech Laboratories, P.O. Box 9364, Kampala, Uganda; ^4^Department of Biosecurity, Ecosystem and Veterinary Public Health, College of Veterinary Medicine, Animal Resources and Biosecurity, Makerere University, P.O. Box 7062, Kampala, Uganda

## Abstract

Sporadic* Salmonella* outbreaks with varying clinical presentations have been on the rise in various parts of Uganda. The sources of outbreaks and factors underlying the different clinical manifestation are curtailed by paucity of information on* Salmonella* genotypes and the associated virulence genes. This study reports molecular diversity of* Salmonella enterica* and their genetic virulence profiles among human and animal isolates. Characterization was done using Kauffman-White classification scheme and virulence genes analysis using multiplex PCR. Overall, 52% of the isolates belonged to serogroup D, 16% to serogroup E, 15% to poly F, H-S, and 12% to serogroup B. Serogroups A, C1, and C2 each consisted of only one isolate representing 5%. Virulence genes located on SPI-1 [*spaN* and* sipB*] and on SPI-2* [spiA]* in addition to* pagC* and* msgA* were equally distributed in isolates obtained from all sources. Plasmid encoded virulence gene* spvB* was found in <5% of isolates from both human epidemic and animal origins whereas it occurred in 80% of clinical isolates. This study reveals that serogroup D is the predominant* Salmonella* serogroup in circulation and it is widely shared among animals and humans and calls for joint and coordinated surveillance for one health implementation in Uganda.

## 1. Introduction

*Salmonella*, subspecies* enterica*, is an important food-borne pathogen responsible for disease in animals and humans. It has been the leading cause of many outbreaks and infections around the world and is considered as one of the major causes of human gastroenteritis worldwide [1]. Animals have been implicated as important sources of* Salmonella*-contaminated food products that are responsible for human salmonellosis and in the United States approximately 40,000 cases of salmonellosis are reported resulting in 600 deaths [1, 2].

The clinical outcome of* Salmonella* infection in humans presents in two broad features; first, it manifests as a serious systemic infection (enteric fever) caused mainly by* Salmonella enterica* serovar Typhi (typhoid fever) and the second usually takes the form of a self-limiting food poisoning (gastroenteritis) and is caused by a large number of nontyphoidal* Salmonella* serovars (NTS) resulting in a range of clinical syndromes including diarrheal disease [3] and fever frequently occurs in developing countries and affects an estimated 21.5 million persons annually, spreading mainly from person to person. In contrast, nontyphoidal salmonellosis is a worldwide disease of humans and animals and has been long recognized as a public health problem responsible for an estimated 1.4 million cases/year, with ~16,000 hospitalizations and 600 deaths in developed countries [4, 5]. In Sub-Saharan Africa, nontyphoidal* Salmonella* are emerging as a prominent cause of invasive disease in infants and young children, although estimates of incidences have been carried out in isolation, giving no overall demographic picture [6].

Notably, the severity of the infection and whether it remains localized in the intestine or disseminates to the bloodstream depend on the* Salmonella* serotype, immune status of the host, and the virulence of the* Salmonella* isolate [7]. The recognition of the virulence in* Salmonella* as a major determinant of the outcome of human infection has been well appreciated [7], and among the various known virulence factors, studies indicated that* Salmonella* species which putatively possess virulence genes such as* hilA* and* invA* are consistently associated with severe illness compared with those which lack such genes [8, 9].

Recently, there have been increased frequencies of sporadic and epidemic* Salmonella* outbreaks associated with high prevalence of intestinal perforation in humans in Uganda [10]. Although the common* Salmonella* serotypes circulating in Uganda and their antibiograms had been fairly studied [11, 12], information regarding the casual relationship between the human-animal overlap of* S. enterica serovars* or their virulence profiles had remained scarce and subsequently warranted an investigation. We hereby report the circulating* Salmonella* serotypes and virulence-associated genes in samples obtained from both humans and animals sources.

## 2. Materials and Methods

### 2.1. Study Design, Population, and Sampling

The study utilized archived* Salmonella* isolate cultures preserved in 20% glycerol (v/w) soft agar at −80°C from both human and animal specimen obtained between 2007 and 2009. The human isolates were obtained from patients diagnosed with gastroenteritis from three sources: Mulago National Referral Hospital, Tororo Hospital, and Kasese District. The human isolates obtained from stool samples or blood cultures of patients with sporadic gastroenteritis visiting Mulago National Referral Hospital and Tororo Hospital from 2007 to 2009 are hereafter referred to as sporadic human clinical isolates. Additional* Salmonella* isolates from Kasese District were collected during an epidemic in the same period, here referred to as human epidemic outbreaks isolates. The animal isolates were collected from rectal faeces in slaughter cattle at the Kampala city abattoir and poultry anal swabs obtained from different geographical places in Kampala city between 2007 and 2009 and archived at the Microbiology Laboratory, College of Veterinary Medicine, Animal Resources and Biosecurity, Makerere University. The study employed census method of sample size estimation. Due to the number of individuals targeted, the selection of* Salmonella* isolates was done using a nonprobability purposive method [13] of all isolates collected from 2007 to 2009. A total of 69* Salmonella* isolates were used in this study, 48 were human isolates, and 21 were animal isolates.

### 2.2. Recovery and Identification of* Salmonella*

Archived bacterial samples were thawed and inoculated into sodium thioglycolate broth using sterile inoculating loop, incubated overnight at 37°C, growth detected by observing turbidity. All further procedures like enrichment, subculturing, and biochemical identification of* Salmonella *were done using standardized selective and identification media [14]. The identified* Salmonella *isolates were then used in subsequent analysis.

### 2.3. Serogrouping of* Salmonella* Isolates

Using commercial monovalent and polyvalent* Salmonella* O antisera (Statens Serum Institute, Denmark), serogrouping was carried out by slide agglutination tests according to the manufacturer's instruction manual. Approximately 10–15 *μ*L of diluted antisera was delivered onto a glass slide. An equal volume of bacterial suspension was added onto the slide and mixed with a loop. The slide was rocked back and forth for 60 seconds while observing agglutination. An isolate was considered to be positive if agglutination occurred. The* Salmonella* isolates were classified into O serogroups based on surface antigens [15]. Polyvalent group F, H-S were isolates that reacted positive to polyvalent O* Salmonella* A-S group antigen but negative to monovalent specific O antigen of serogroups A, B, C1, C2, D, E, and G.

### 2.4. Antimicrobial Susceptibility Testing of Isolates

The antibiotic susceptibility profiling of the* Salmonella* isolates was determined by Kirby-Bauer disk diffusion method [16]. Seven different antibiotics were used in this study, namely, tetracycline (30 *μ*g), chloramphenicol (30 *μ*g), trimethoprim-sulphamethoxazole (25 *μ*g), ciprofloxacin (5 *μ*g), nalidixic acid (30 *μ*g), ampicillin (10 *μ*g), and ceftriaxone (30 *μ*g) (Mast Group Ltd, Merseyside, UK). Antimicrobial susceptibility was done on Muller Hinton agar (Mast Group Ltd, Merseyside, UK) and* Escherichia coli* ATCC 25329 control strain was used. The diameter of zonal clearance was measured in millimeters and results were recorded as susceptible (S), intermediate (I), and resistant (R) according to CLSI, performance standards for antimicrobial susceptibility testing [17].

### 2.5. Molecular Analysis

#### 2.5.1. Total DNA Extraction and Purification

Genomic DNA from* Salmonella* isolates cultured in a sodium thioglycolate broth was extracted using the chloroform-isoamyl alcohol method. Briefly, Cells were harvested by centrifugation at 2400 g for 10 min in an IEC CL31R multispeed centrifuge (Thermo Scientific). The supernatant was discarded and the pellet resuspended in 400 *μ*L Tris-EDTA (TE) buffer, containing 0.01 M Tris-HCl pH 7.4, 0.001 M EDTA with gentle agitation. Seventy microliters of 10% SDS (sodium dodecyl sulphate) (Fisher Scientific) was added to the suspension, followed by 5 *μ*L of (10 mg/mL stock solution) proteinase K (Ambion®). The samples were then incubated for 1 hr at 65°C in hybridization oven (Biometra OV2, Anachem, UK). After incubation, 100 *μ*L of 5 M NaCl followed by 100 *μ*L CTAB/NaCl (prewarmed at 70°C) was added to the solutions. The solution was gently inverted for 10 sec, incubated at 65°C for 20 min, and then cooled at room temperature for 5 min. Seven hundred and fifty microliters of chloroform-isoamyl alcohol (24 : 1, Sigma-Aldrich) was added and centrifuged at 1300 ×g for 15 min. The supernatants were transferred to new tubes, treated with 5 *μ*L RNase A (5 mg/mL in RNase A buffer containing 0.5 M NaCl, 0.01 M EDTA), and incubated at 37°C for 30 min. DNA was precipitated with 500 *μ*L ice-cold isopropanol containing 0.1 volume sodium acetate and mixed carefully by inverting the tubes. The pellets were incubated at −80°C for 30 min and centrifuged at 13000 ×g for 10 min. The supernatant was discarded and the pellets were washed twice with 1 mL of cold 70% ethanol followed by centrifugation at 13000 ×g for 15 min. The pellets of DNA were dried at 37°C for 30 min and reconstituted in 50 *μ*L elution buffer [Qiagen]. The quality of DNA was examined by running 4 *μ*L of the DNA sample on 0.85% agarose gel. The DNA was subsequently stored at −20°C awaiting further analysis.

#### 2.5.2. Multiplex PCR for Detection of Virulence Genes

In order to predict the virulence potential of* Salmonella* isolates from different sources, the presence of 6 virulence genes (*spiA, pagC, msgA, sipB, spaN,* and* spvB*) was screened as two runs; that is, run 1 consisted of 4* (spiA, pagC, msgA, sipB)* and run 2 consisted of 2 (*spaN* and* spvB*) genes, respectively, using multiplex PCR assays.

The forward and reverse primers used for detection of virulence gene and the amplification conditions were adapted from Versalovic et al. [18]. Amplifications were performed in a 25 *μ*L reaction mixture comprising 2.5 *μ*L of template DNA, 16.3 *μ*L of RNase free H_2_O, 2.5 *μ*L of 10x PCR buffer, 3.0 *μ*L of 50 mM MgCl_2_, 0.15 *μ*L of Taq 5 U/*μ*L [Thermo Fisher], 0.5 *μ*L of 10 mM dNTPs mix (New England Biolabs®), and 0.05 *μ*L of 0.1 mM forward and reverse primers. Amplification was performed in a thermocycler (MJ Research, PTC 200 USA) under the following conditions: 1 cycle of initial denaturation at 95°C for 5 min, denaturation at 94°C for 30 sec, and annealing for 1 min at 53°C for* spvB, spiA, pagC, *and* msgA* and at 56°C for* sipB *and* spaN* and 2 min at 72°C, with a final cycle of 10 min at 72°C, followed by a hold at 4°C. The resulting PCR products were subjected to horizontal gel electrophoresis in 2% agarose and visualized using BioDoc-It™ Imaging (UVP, USA) and images retrieved from the computer in the data office.

### 2.6. Data Analysis

Virulence gene results were analyzed visually and an isolate was considered to contain the virulence gene of interest if it produced an amplicon of the expected size as judged by the molecular marker. These scores were entered into a Microsoft Excel 2007 spreadsheet and then tabulated.

## 3. Results

### 3.1. General Serogroup Distribution Pattern of* Salmonella* Isolates Obtained from Different Sources

**Figure 1:**
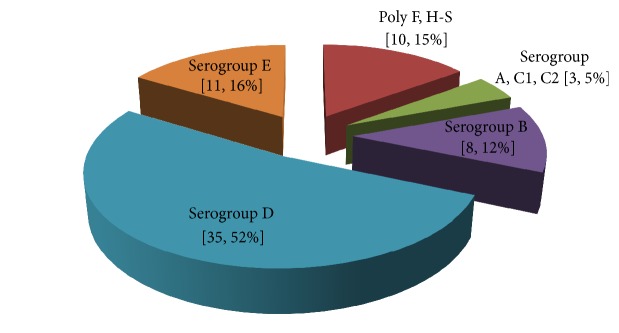
General serogroup distribution pattern of* Salmonella* isolates obtained from different sources.

The 69* Salmonella* isolates used in this study were obtained from three sources: 25 (36%) from sporadic human clinical cases, 23 (34%) from human epidemic outbreaks, and 21 (30%) from animal sources (Table 1). A total of 67 isolates were typable using polyvalent O antisera for A-S antigens; the nontypable O antigen was expressed by 2 isolates which were recorded as NT as shown in Table 1. Further subtyping of 67 polyvalent A-S positive isolates segregated the isolates into serogroups A, B, C1, C2, D, E, and G. The monovalent specific serotyping indicated that majority of isolates (52%) belonged to serogroup D, serogroup E 16%, poly F, H-S 15%, and serogroup B with 12% (Figure 1). The remaining serogroups A, C1, and C2 each consisted of only one isolate representing 5% of the isolates (Table 1). Of the 10 isolates belonging to poly F, H-S, 6 (60%) were of human clinical origin and 40% were of animal origin. The animal isolates were obtained from pigs, 2 (9.7%), cattle, 7 (33.3%), and chickens, 12 (57%), as in Table 2. All isolates belonging to serogroups A, B, C1, C2, and E were exclusively of human clinical and animal origins, respectively. Of the 35 isolates belonging to group D, the majority, 63%, were from human epidemic followed by 23% from human clinical cases and 14% from animal origins as shown in Table 1. Overall, the majority of the human isolates (30 of 48; 62%) belonged to serogroup D (Table 1). With respect to the source, 22 of 23 (96%) isolates from human epidemic cases belonged to group D (Table 1). While one isolate was nontypable, none of the* Salmonella* belonging to serogroups A, B, C1, C2, and E were detected (Table 3). On the other hand, isolates obtained from sporadic human infections, 35% (8 of 23), belonged to serogroup B or D, while 6 of 23 (26%) were poly F, H-S (Table 1). The remaining 3 isolates each belonged to serogroups A, C1, and C2, respectively. None of isolates in serogroup E or expressing a nontypable O antigen were detected.

### 3.2. Prevalence of Serogroups of* Salmonella* from Different Species of Animals

The majority of the isolates, 11 of 21 (52.4%), were of serogroup E, followed by 5 of 21 (24%) for serogroup D, while 19% belonged to polyvalent F, H-S group (Table 2). One isolate expressing a nontypable O antigen was detected. None of isolates in serogroups A, B, C1, C2, and G were detected. Of the 3 animal species from which specimens were taken, chicken had all the three serogroups (E, D, and poly F, H-S) with 6 of 12 (50%) from serogroup E, 4 of 12 (33%) from serogroup D, and the rest from group F, H-S (Table 2). Similarly, 4 out of 7 (57%) of isolates from cattle belonged to serogroup D. One isolate belonged to serogroups D and E each in the pig isolates (Table 2).

### 3.3. Antimicrobial Resistance Profiling

To understand the antimicrobial resistances profiles of the* Salmonella* under this study, all 69 isolates were tested against seven antibiotics (Table 3). The antimicrobial drugs used were ciprofloxacin (Cip), ceftriaxone (CRO), nalidixic acid (NA), chloramphenicol (C), trimethoprim-sulphamethoxazole (SXT), ampicillin (Amp), and tetracycline (T). All* Salmonella* serogroups and nontypable isolates showed no resistance to ciprofloxacin and ceftriaxone while 12.5% resistance to nalidixic acid was detected. Serogroup A isolate was resistant to all antibiotics assayed (Table 3).

However, resistance pattern to the rest of the antibiotics varied widely among serogroups. Serogroup D showed the highest variability from 20% to 97% resistance to chloramphenicol, tetracycline, trimethoprim-sulphamethoxazole, and ampicillin antibiotics. All the isolates of serogroups C1 and C2 were susceptible to all the antibiotics assayed except ampicillin. The resistance pattern for polyvalent group F, H-S ranged from 50% to 80% for tetracycline, trimethoprim-sulphamethoxazole, and ampicillin. Isolates belonging to serogroup B showed more than 50% resistance to all the above four antibiotics while isolates of group E showed resistance to ampicillin and tetracycline. From this study,* Salmonella* isolates showed the highest resistance to ampicillin followed by trimethoprim-sulphamethoxazole. The most resistant serogroups were A and B and these were predominantly found in human clinical specimens (Table 3).

**Figure 2:**
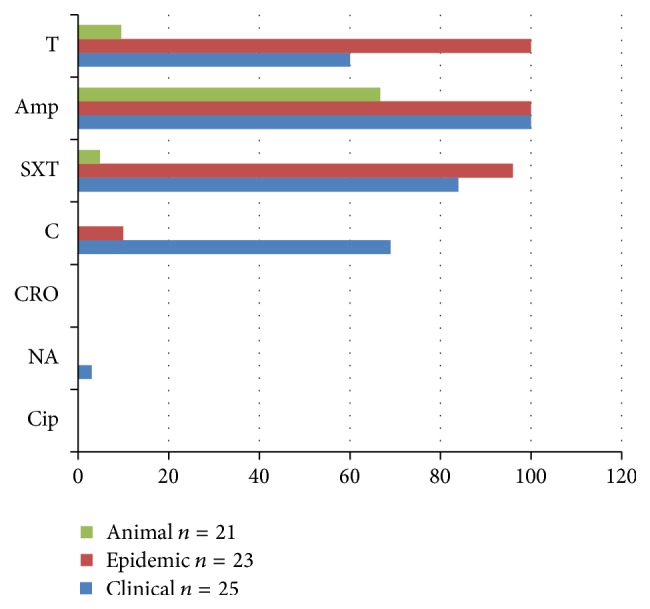
A bar graph showing resistance patterns of* Salmonella* isolates according to origins. NA = nalidixic acid; Cip = ciprofloxacin; CRO = ceftriaxone; C = chloramphenicol; SXT = trimethoprim-sulphamethoxazole; Amp = ampicillin; T = tetracycline.

Over 60% of all the isolates were resistant to trimethoprim-sulphamethoxazole, ampicillin, and tetracycline (Figure 2). The antibiotic susceptibility test revealed the presence of multiple drug resistance in both routine clinical and epidemic isolates. Furthermore, human clinical isolates showed the highest variability in resistance patterns from 60% to 100% for all antibiotics from tetracycline to ampicillin (Figure 2). Isolates obtained from human epidemic cases registered >95% resistance to 3 antibiotics (trimethoprim-sulphamethoxazole, tetracycline, and ampicillin) and a relatively low resistance to chloramphenicol at 9% compared to human clinical cases isolates. On the other hand, animal* Salmonella* isolates showed minimal resistance of less than 10% to trimethoprim-sulphamethoxazole and tetracycline but a higher resistance of 67% to ampicillin (Figure 2). All isolates were susceptible to ceftriaxone and ciprofloxacin. One isolate of clinical origin showed resistance to nalidixic acid.

### 3.4. Distribution of Virulence Genes among* Salmonella* Isolates of Different Origins

**Figure 3:**
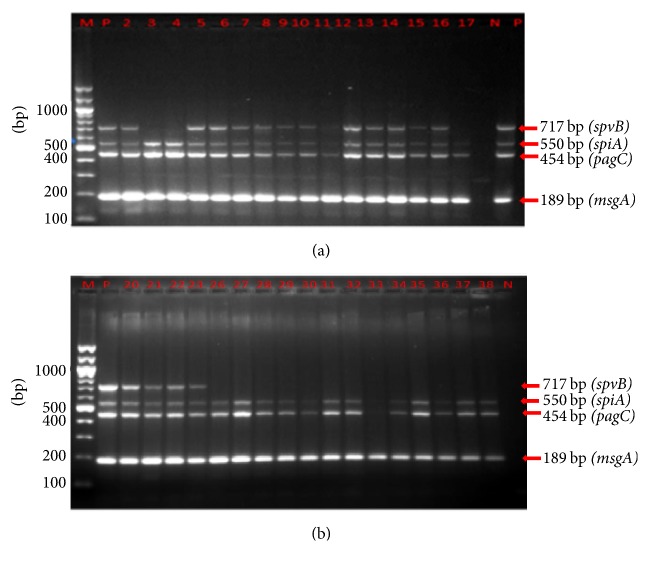
Multiplex PCR results for* spvB, sipA, pagC, *and* msgA* genes in human* Salmonella* isolates. (a) M, 100 bp ladder; P, positive control; N, negative control; lanes 2–17, human clinical isolates. (b) Lane M, 100 bp ladder; P, positive control; N, negative control; lanes 18–21, human clinical isolates from sporadic cases; lanes 22–34,* Salmonella* isolates from human epidemic cases.

**Figure 4:**
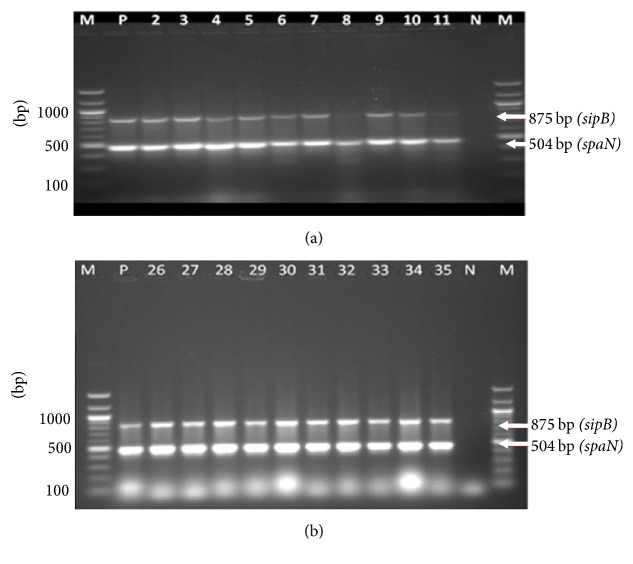
Multiplex PCR results for* sipB and spaN genes* in human* Salmonella* isolates. (a) Lane M, 100 bp ladder; P, positive control; N, negative control; lanes 2–11, human clinical isolates. (b) Lane M, 100 bp ladder; P, positive control; N, negative control; lanes 26–35,* Salmonella* isolates from human epidemic outbreaks.

**Figure 5:**
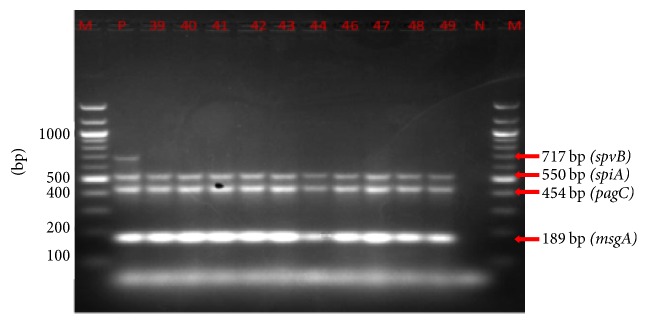
Multiplex PCR results for* spvB, sipA, pagC,* and* msgA* genes in animal* Salmonella* isolates. Lane M, 100 bp ladder; P, positive control; N, negative control; lanes 39–49, samples.

As shown in Figures 3(a) and 3(b) and Table 4, majority of* Salmonella* isolates carried* spiA, pagC, *and* msgA *genes regardless of origin. In contrast,* spvB* was only detected in 1 out of 21 (5%) isolates from the epidemic and in 80% of the sporadic cases (Table 4). Further amplification of two virulence genes* (spaN and sipB)* was carried out and the resultant banding pattern is shown in Figures 4(a) and 4(b). Overall, the most prevalent virulence-associated genes in human epidemic cases were* msgA* and* spaN* while* spiA*,* sipB*, and* pagC* were present in about 80% of the isolates (Table 4, Figures 3(b) and 4(b)). Multiplex PCR assays for the amplification of (*spvB, spiA, pagC*,* msgA, sipB,* and* spaN*) genes were carried out in* Salmonella* isolates from the animal samples (Figure 5). Isolates of animal origin exhibited the same virulence-associated gene pattern as those of human origin (Table 4 and Figure 5). Isolates from cattle (>85%) possessed* spaN, msgA, sipB, pagC, *and* spiA *and only one had* spvB *(Table 4). Strikingly,* SpvB* was not found in isolates from either pig or poultry. Overall, there appeared to be no major differences in the virulence-associated gene profiles of animal (poultry, cattle, and pig) and outbreak sources, or any differences among the multiple isolates of human origin (Table 4).

### 3.5. Carriage of Virulence Genes by* Salmonella* Isolates from Different Serogroups

The distribution of the 6 virulence-associated genes in* Salmonella* is summarized as shown in Table 5. All the virulence genes assayed except* spvB* were present in all the isolates belonging to serogroups A, B, C1, and C2 and in more than 80% of isolates belonging to serogroups D and E.* spvB* was present in 7 (87.5%) of the isolates in serogroup B while being found in all the isolates belonging to serogroups A and C1. Conversely, none of the isolates in serogroup C2 possessed* spvB* and only 9 (25.7%) isolates belonging to serogroup D had this virulence gene.* spvB* was found in 9.7% and 40% of isolates belonging to serogroups E and polyvalent F, H-S, respectively. All the 11 isolates in serogroup E had* sipB *and* spaN* whereas 10 had* msgA, pagC, *and* spiA*. About 60% of isolates in polyvalent F, H-S had* spiA, pagC, *and* sipB* while 7 (70%) had* msgA* and* spaN* with 4 (40%) having* spvB*. Interestingly, none of the 2 nontypable isolates had any of the six virulence genes assayed in this study.

## 4. Discussion

This study sought to characterize* Salmonella enterica* isolates from both animal and humans samples as well as detecting the associated virulence genes. Phenotyping showed a high variation in serogroups among isolates obtained from all the sources. Fifty-two percent of all the isolates belonged to serogroup D, 16% to serogroup E, 12% to serogroup B, and 15% to poly F, H-S while serogroups A, C1, and C2 each consisted of only one isolate. Not surprisingly, all the serogroups identified in this study belong to* Salmonella enterica* I subspecies which is responsible for most of human salmonellosis [19]. There was a remarkable variation in distribution of serogroups according to isolate sources. Of the 35 isolates belonging to serogroup D, majority, 63%, were from human epidemic cases, 23% from routine clinical cases, and 14% from animal origins. This indicates that there is wide sharing of isolates between animals and humans, incriminating animals partly as potential source of clinical salmonellosis. Previous studies have highlighted the significance of zoonotic salmonellosis in human infections [20]. It was observed that* Salmonella* isolates collected from the human epidemic used in this study were serotyped as* S. Typhi* [10]. The fact that* S. Typhi* is not animal adapted [21] implies that the animal isolates that shared serogroup D could be of different serotype. This is because the antigenic coat D is shared by diverse* Salmonella* strains belonging to multiple serotypes.

Conversely, isolates belonging to serogroup E were exclusively of animal origin and this result conforms to existing literature showing that all serotypes in serogroup E are animal restricted [22]. One striking observation is that most of the human clinical salmonellosis was caused by isolates of divergent serogroups (A, B, C1, and C2) not found in samples from animal origin with exception of poly F, H-S, suggesting that the potential source of clinical infection could have been from the environment, soil, and water. However, the role of animals as potential source of these pathogens could not be ruled out since a small sample size was used. There is need for a larger sample size in future studies to determine whether such serogroups reported in this study may be infecting animals in Uganda.

In terms of the distribution of drug resistance among* Salmonella* isolates, high variability of patterns with respect to serogroups and sources is confirmed. Although animals are known to be the main reservoir of* Salmonella* and a common source of human contamination [1, 2], it is interesting to note that the frequency of antibiotic resistance to tetracycline, ampicillin, and trimethoprim-sulphamethoxazole in this study was higher in isolates of human origin than in animal and/or poultry isolates. This observation conflicts with reports by other authors [23, 24] and extricates the antibiotic use in animal feeds as a potential source of resistance induction to human infections. Plausible explanations could be as follows: (i) selective pressure existing in hospital environments that strongly contribute to the increasing resistance of strains isolated in such environments; (ii) irrational use of antibiotics through either easy access self-medication, poor prescription, or incomplete dosage in the general population. The same reasons could also shed light on the observed resistance to chloramphenicol and nalidixic acid exhibited by the clinical isolates collected from Kampala versus epidemic samples from Kasese District and further corroborated findings by Mahero et al., 2013 [24]. Although these isolates belonged to a different serogroup from those causing human epidemics, this poses a big threat to public health interventions and calls for effective surveillance program to ascertain its magnitude.

*Salmonella* isolates incriminated in human epidemic outbreak, clinical cases, and* Salmonella *isolated from clinically healthy animals were assayed for their possession of certain putative virulence genes in an effort to identify factors that are important to the virulence of this organism. Of particular interest in this study were genes involved in intracellular survival, adhesion, and invasiveness [25, 26]. The genes include* spaN*,* sipB,* and* spiA* which are associated with type III secretory system [27–29]. The* msgA*,* spvB,* and* pagC* genes encode products that are associated with cell invasion, survival within the cell, and adhesin or pili production [30–32].

Genes located on SPI-1 (*spaN* and* sipB*), and on SPI-2* (spiA)* along with* pagC* and* msgA* were found in 100% of isolates belonging to serogroups A, C1, C2, and B and in more than 80% in isolates in serogroups D and E, suggesting a ubiquity of these genes in* Salmonella* serogroups. Similarly, these genes were present in over 80% of isolates obtained from either human clinical, epidemic, or animal origins suggesting their wide distribution among* Salmonella* isolates regardless of their host of origin. A recent study assaying >50 genes also did not find major differences in the virulence-associated gene profiles of the poultry (egg houses and farm) and outbreak strains, nor any differences among the multiple isolates of serovars [33]. Similarly, another study, which included 17 genes, did not find any significant difference between sick and clinically healthy birds [34].

In contrast to these highly prevalent genes,* spvB* gene was found in <5% and 15% of isolates from human epidemic and animal origins, respectively, whereas it occurred in about 80% of clinical isolates. In addition, this gene was present in >80% of isolates belonging to serogroups A, B, and C1 and in <23% of isolate in serogroups D, E, and poly F, H-S. However, not all isolates of a plasmid bearing serogroups contain this virulence plasmid, which could explain why* spvB* was found in such a low proportion of isolates compared with chromosomal genes. Overall, the strong similarities in virulence genotypes between isolates obtained from different origins might indicate that the* Salmonella* of animal and human clinical and epidemic cases are capable of causing salmonellosis under conditions conducive to illness and that virulence genotyping, at least with genes studied here, might have marginal influence in enhancing severe infection. This study also reveals that serogroup D is the predominant* Salmonella* serogroup in circulation and it is widely shared among animals and humans and calls for a joint and coordinated surveillance for one health implementation in Uganda.

To the best of our knowledge, the present study is the first comprehensive study attempting to provide insight into the genetic diversity of* Salmonella* and its associated virulence genes circulating in both humans and animals in Uganda.

## Figures and Tables

**Table 1:** Distribution of *Salmonella* isolates obtained from different origins according to serogroups.

Sources of isolates				
Serogroups	Human clinical (*n* = 25)	Human epidemic (*n* = 23)	Animal	Total
			(*n* = 21)	*n* = 69
Poly F, H-S	6	0	4	10
A	1	0	0	1
B	8	0	0	8
C1	1	0	0	1
C2	1	0	0	1
D	8	22	5	35
E	0	0	11	11
Untypable	0	1^*∗*^	1^*∗*^	2

All isolates were negative to monovalent O antisera G serogrouping.  ^*∗*^Untypable isolates.

**Table 2:** Prevalence of *Salmonella* serogroups from different species of animals.

Serogroup	Chicken	Cattle	Pig	Total
	*n* = 12	*n* = 7	*n* = 2	*N* = 21
Poly F, H-S	4	0	0	4
D	2	2	1	5
E	6	4	1	11
Nontypable	0	1	0	1

None of isolates in serogroups A, B, C1, and C2 were detected.

**Table 3:** Antibiotic resistance profiles of *Salmonella* isolates according to serogroups.

*Antibiotics tested*	*N* (%) of resistant *Salmonella* serogroups against selected antibiotics					
	B	C1	C2	D	E	F, H-S
	(*n* = 8)	(*n* = 1)	(*n* = 1)	(*n* = 35)	(*n* = 11)	(*n* = 10)
C (30 *μ*g)	6 (75)	0	0	07 (20)	0	5 (50)
SXT (25 *μ*g)	6 (75)	0	0	29 (83)	0	6 (60)
Amp (10 *μ*g)	8 (100)	1 (100)	1 (100)	34 (97)	7 (66)	8 (80)
T (30 *μ*g)	4 (50)	0	0	27 (77)	1 (9)	5 (50)

**Table 4:** Distribution of virulence-associated genes among *Salmonella* isolates according to origins.

Virulence genes	Number (%)					
	Clinical samples	Human epidemic	Cattle	Pigs	Poultry	All samples
	*n* = 25	*n* = 23	*n* = 7	*n* = 2	*n* = 12	*N* = 69
*spvB*	20 (80)	01 (4.3)	01 (14.3)	00 (0.0)	00 (0.0)	22 (31.9)
*spiA*	22 (88)	19 (82.6)	06 (85.7)	01 (50.0)	08 (66.7)	56 (81.0)
*pagC*	23 (92)	19 (82.6)	06 (85.7)	01 (50.0)	08 (66.7)	57 (82.6)
*msgA*	23 (92)	22 (95.6)	06 (85.7)	02 (100)	09 (75.0)	62 (89.9)
*sipB*	21 (84)	20 (87.0)	06 (85.7)	02 (100)	10 (83.3)	59 (85.5)
*spaN*	23 (92)	22 (95.6)	06 (85.7)	02 (100)	10 (83.3)	63 (91.3)

**Table 5:** Distribution of virulence-associated genes among predominant *Salmonella* serogroups.

Virulence genes	Number (%) of isolates per serogroup positive for the different virulence genes		
	D (*n* = 35)	E (*n* = 11)	F, H-S (*n* = 10)
*spvB*	8 (22.8)	1 (9.1)	4 (40)
*spiA*	29 (82.9)	10 (90.9)	6 (60)
*pagC*	30 (85.7)	10 (90.9)	6 (60)
*msgA*	34 (97.1)	10 (90.9)	7 (70)
*sipB*	30 (85.7)	11 (100)	6 (60)
*spaN*	34 (97.1)	11 (100)	7 (70)

## References

[1] Scallan E., Angulo F. J., Kirk M. (2010). The global burden of nontyphoidal *Salmonella* gastroenteritis. *Clinical Infectious Diseases*.

[2] CDC (2005). Salmonellosis associated with pet turtles-Wisconsin and Wyoming. *Morbidity and Mortality Weekly Report (MMWR)*.

[3] Miller S. I., Pegues D. A., Madell G. L., Bennett J. E., Dolin R. (2000). *Salmonella* species, including *Salmonella* typhi. *Principles and Practice of Infectious Disease*.

[4] Adak G. K., Long S. M., O'Brien S. J. (2002). Trends in indigenous foodborne disease and deaths, England and Wales: 1992 to 2000. *Gut*.

[5] Voetsch A. C., Van Gilder T. J., Angulo F. J. (2004). Food-Net estimate of the burden of illness caused by nontyphoidal *Salmonella* infections in the United States. *Clinical Infectious Diseases*.

[6] Tennant S. M., Diallo S., Levy H. (2010). Identification by PCR of non-typhoidal *Salmonella enterica* serovars associated with invasive infections among febrile patients in Mali. *PLoS Neglected Tropical Diseases*.

[7] Hook E. W. (1961). Salmonellosis: certain factors influencing the interaction of Salmonella and the human host. *Bulletin of the New York Academy of Medicine*.

[8] Lesnick M. L., Reiner N. E., Fierer J., Guiney D. G. (2001). The *Salmonella* spvB virulence gene encodes an enzyme that ADP-ribosylates actin and destabilizes the cytoskeleton of eukaryotic cells. *Molecular Microbiology*.

[9] Castagna S. M. F., Muller M., Macagnan M., Rodenbusch C. R., Canal C. W., Cardoso M. (2005). Detection of *Salmonella* sp. from porcine origin: a comparison between a PCR method and standard microbiological techniques. *Brazilian Journal of Microbiology*.

[10] Neil K. P., Sodha S. V., Lukwago L. (2012). A large outbreak of typhoid fever associated with a high rate of intestinal perforation—Kasese district, Uganda, 2008-2009. *Clinical Infectious Diseases*.

[11] Ikwap K., Erume J., Owiny D. O. (2014). Salmonella species in piglets and weaners from Uganda: prevalence, antimicrobial resistance and herd-level risk factors. *Preventive Veterinary Medicine*.

[12] Afema J. A., Byarugaba D. K., Shah D. H., Atukwase E., Nambi M., Sischo W. M. (2016). Potential sources and transmission of *Salmonella* and antimicrobial resistance in Kampala, Uganda. *PLoS ONE*.

[13] Dolores M., Tongco C. (2007). Purposive sampling as a tool for informant selection. *Ethnobotany Research & Applications*.

[14] World Health Organisation Manual for the laboratory identification and antimicrobial testing of bacterial pathogens of public health importance in developing world.

[15] Kauffman G. (1974). Kauffman White Scheme. WHO. Pd 172, 1, rev. 1. *Acta Pathologica et Microbiologica Scandinavica Section B-Microbiology*.

[16] Sherris J. C., Turck M. (1966). Antibiotic susceptibility testing by a standardized single disk method. *American Journal of Clinical Pathology*.

[17] Clinical and Laboratory Standard Institute (CLSI) (2010). Performance standards for antimicrobial susceptibility testing; 20th informational supplement (June 2010, update). *CLSI Document*.

[18] Versalovic J., Schneider M., de Bruijn F. J., Lupski J. R. (1994). Genomic fingerprinting of bacteria using repetitive sequence based PCR (rep-PCR). *Methods in Molecular Cell Biology*.

[19] Brenner F. W., Villar R. G., Angulo F. J., Tauxe R., Swaminathan B. (2000). Salmonella nomenclature. *Journal of Clinical Microbiology*.

[20] Alcaine S. D., Soyer Y., Warnick L. D. (2006). Multilocus sequence typing supports the hypothesis that cow- and human-associated *Salmonella* isolates represent distinct and overlapping populations. *Applied and Environmental Microbiology*.

[21] Morrow B. J., Graham J. E., Curtiss R. (1999). Genomic subtractive hybridization and selective capture of transcribed sequences identify a novel *Salmonella* typhimurium fimbrial operon and putative transcriptional regulator that are absent from the *Salmonella* typhi genome. *Infection and Immunity*.

[22] CDC (2006). *Salmonella Surveillance: Annual Summary*.

[23] Bacci C., Boni E., Alpigiani I., Lanzoni E., Bonardi S., Brindani F. (2012). Phenotypic and genotypic features of antibiotic resistance in *Salmonella enterica* isolated from chicken meat and chicken and quail carcasses. *International Journal of Food Microbiology*.

[24] Mahero M., Byarugaba D. K., Doetkott D. K., Olet S., Khaitsa M. L. (2013). Antimicrobial resistance and presence of class 1 integrons in *Salmonella* serovars isolated from clinical cases of animals and humans in North Dakota and Uganda. *Clinical Microbiology*.

[25] Nolan L. K., Wooley R. E., Brown J., Payeur J. B. (1991). Comparison of phenotypic characteristics of *Salmonella* spp isolated from healthy and ill (infected) chickens. *American Journal of Veterinary Research*.

[26] Kottom T. J., Nolan L. K., Brown J. (1995). Invasion of Caco-2 cells by *Salmonella* Typhimurium (Copenhagen) isolates from healthy and sick chickens. *Avian Diseases*.

[27] Ochman H., Soncini F. C., Solomon F., Groisman E. A. (1996). Identification of a pathogenicity island required for *Salmonella* survival in host cells. *Proceedings of the National Academy of Sciences*.

[28] Suarez M., Russmann H. (1998). Molecular mechanisms of *Salmonella* invasion: the type III secretion system of the pathogenicity island 1. *Internatl Microbiol*.

[29] Waterman S. R., Holden D. W. (2003). Functions and effectors of the *Salmonella* pathogenicity island 2 type III secretion system. *Cell Microbiol*.

[30] Groisman E. A., Ochman H. (1997). How *Salmonella* became a pathogen. *Trends in Microbiology*.

[31] Van der Velden A. W. M., Baumler A. J., Tsolis R. M., Heffron F. (1998). Multiple fimbrial adhesins are required for full virulence of *Salmonella* typhimurium in mice. *Infection and Immunity*.

[32] Haghjoo E., Galan J. E. (2004). Salmonella typhi encodes a functional cytolethal distending toxin that is delivered into host cells by a bacterial internalization pathway. *Proceedings of the National Academy of Sciences*.

[33] Zou W., Al-Khaldi S. F., Branham W. S. (2011). Microarray analysis of virulence gene profiles in *Salmonella* serovars from food/food animal environment. *Journal of Infection in Developing Countries*.

[34] Jerod A. S., Catherine M. L., Lisa K. N. (2006). Virulence genotyping of *Salmonella* spp. with multiplex PCR. *Avian Diseases*.

